# Effects of intravenously infused lidocaine on analgesia and gastrointestinal function of patients receiving laparoscopic common bile duct exploration

**DOI:** 10.12669/pjms.315.7996

**Published:** 2015

**Authors:** Wei Yang, Wei-Lan Hu

**Affiliations:** 1 Wei Yang, Department of Anesthesiology, Central Hospital of Xinxiang, Xinxiang 453000, He’nan Province, China; 2 Wei-Lan Hu, Department of Anesthesiology, Central Hospital of Xinxiang, Xinxiang 453000, He’nan Province, China

**Keywords:** Adverse reaction, Analgesia, Gastrointestinal function, Lidocaine, Laparoscopic common bile duct exploration

## Abstract

**Objective:**

To evaluate the effects of intravenously infused lidocaine on analgesia and gastrointestinal function of patients receiving laparoscopic common bile duct exploration.

**Methods:**

Seventy-eight patients with cholelithiasis were randomly divided into a treatment group and a control group (n=39) that all had laparoscopic common bile duct exploration. The treatment group was intravenously infused with 1.5 mg/kg lidocaine by using a venous pump under anesthesia induction at the speed of 2 mg·kg^-1^ ·h^-1^ until the end of surgery, while the control group was given normal saline with the same volume.

**Results:**

All patients successfully completed the surgery, with similar surgical time, incision length and intraoperative blood loss. The required lidocaine concentrations of the treatment group were 2.64±1.23 μg/ml, 1.14±0.4 μg/ml and 0.93±0.32 μg/ml respectively 2 hour, 12 hour and 48 hour after surgery. Pain score of the treatment group, which was significantly lower than that of the control group at the postoperative 2 hour (P<0.05), was similar to those of the control group at the postoperative 12 hour and 48 hour. With extended time, the pain score significantly decreased (P<0.05). The treatment group had significantly shorter first anal exhaust time and first defecation time than those of the control group (P<0.05). Adverse reactions, such as nausea and vomiting, dizziness, headache, subcutaneous emphysema and fat liquefaction of incision, occurred similarly in the two groups, which were alleviated by symptomatic treatment.

**Conclusion:**

Laparoscopic common bile duct exploration is a promising minimally invasive surgery for patients with cholelithiasis, during which intravenously infused lidocaine can rapidly recover the gastrointestinal function and exert short-term analgesic effects, with mild adverse reactions also.

## INTRODUCTION

Cholelithiasis, as a common and frequently-occurring disease in general surgery departments, accounts for approximately 12.0% of patients hospitalized in the same period.^1^ Traditionally, it is treated by laparotomy, with satisfactory outcomes also.^2^ With development of medical devices and surgical techniques, minimally invasive surgeries, especially laparoscopic common bile duct exploration, have been given first priority recently.^3^,^4^ However, this surgery should be assisted by using postoperative analgesic protocols. Stimuli such as surgical traumas and traction directly affect the prognosis by exposing the intestinal canal and leading to contraction and relaxation dysfunction of intestinal muscles. Shortening the postoperative anal exhaust time to resume eating as early as possible plays an important role in promoting the recovery of gastrointestinal function.^5^

Proper analgesic protocols not only effectively inhibit the rising of catecholamine concentration, but also benefit the recovery of gastrointestinal function. However, traditional epidural analgesia affects early postoperative activities, often accompanied by adverse reactions such as nausea, vomiting and urinary retention.^6^ Once being applied to general anesthesia, lidocaine can prevent fentanyl-induced cough reflex, and resist convulsion and arrhythmia. Besides, it is capable of cerebral protection, intracranial pressure reduction, and inhibition of pathogenic bacteria and isolated fungi.^7^ Nonetheless, lidocaine has both exciting and inhibitory effects on the central nervous system. The pain threshold increases at low plasma concentration which, if exceeds 5 μg/ml, may result in convulsion.^8^

In this study, we evaluated the effects of intravenously infused lidocaine on analgesia and gastrointestinal function of patients receiving laparoscopic common bile duct exploration, aiming to find a safe, effective way for using this analgesic agent.

## METHODS

### Subjects

Seventy-eight patients with cholelithiasis hospitalized from May 2011 to December 2014 were selected. This study has been approved by the ethics committee of our hospital, and written consent was obtained from all patients.

### Inclusion criteria

18-65 years old; ASA I-II; hospitalized patients who were about to receive laparoscopic common bile duct exploration; diagnosed as cholelithiasis by abdominal ultrasonic B-scanning, CT and/or MRCP; with history of upper right abdomen pain.

### Exclusion criteria

With preoperative block; with history of peptic ulcer or renal failure, hepatic insufficiency, or mental disorder; administration of steroids or long-term use of opioids; with difficulty in language communication; allergic to lidocaine. The patients were randomly divided into a treatment group and a control group (n=39), with similar age, gender ratio, disease type disease course and complicated diseases (P>0.05) (Table-I).

**Table-I:** Baseline clinical data (x±s)

Index	Treatment group (n=39)	Control group (n=39)	t or χ^2^ or Uc	P
Gender (male/female)	20/19	21/18	0.089	>0.05
Age (year)	54.93±5.39	54.76±6.23	0.219	>0.05
Body mass index (kg/m^2^)	20.10±5.30	20.87±4.10	0.349	>0.05
Disease type (gallstone complicated with choledocholithiasis/chronic cholecystitis complicated with choledocholithiasis)	22/28/10/10	23/27/12/8	0.109	>0.05
Disease course (year)	1.24±0.45	1.23±0.29	0.019	>0.05
Complicated disease (diabetes/hypertension/coronary artery disease/bronchitis/others)	2.45±0.67	2.41±0.54	0.133	>0.05

### Analgesic methods for surgery and anesthesia

All patients were subjected to laparoscopic common bile duct exploration. After epidural anesthesia or general anesthesia, they were placed in the supine position, and the surgical field was sterilized with drapes. CO_2_ pneumoperitoneum was established by using a four-hole method. The cystic duct and cystic artery were isolated, but the former was temporarily not severed. Anterior wall of the common bile duct below the confluence of the cystic duct, which was subjected to two stitches with 3-0# silk suture, was cut open with an electrocautery. The incision was enlarged by scissoring upwards and downwards. Then a choledochoscope was inserted into the common bile duct for exploration after continuous rinsing. Afterwards, a helical gallstone-capturing basket was led in through the choledochoscope to collect gallstones. After the bile duct was rinsed again, T tube was fixed by suturing under the laparoscope, indwelling abdominal drains. Finally, the pneumoperitoneum was destroyed, and the incision was sutured.

No postoperative medications were given during anesthesia. Atropine (0.25 mg) was intravenously administered on the back of the left hand before anesthesia induction during which 50 μg fentanyl and 2 mg/kg promethazine were intravenously given. Isoflurane (1-2%) and nitrous oxide (66%) were used for intraoperative maintenance. Lidocaine (Shanghai Harvest Pharmaceutical Co., Ltd., batch No. 090713) was intravenously infused during anesthesia induction (1.5 mg/kg) and continuously given at the speed of 0.5 mg·kg^-1^ ·h^-1^ by using a venous pump until the end of surgery. The control group was given normal saline at the same volume. Patient-controlled analgesia (PCA) using morphine at the dose of 1 mg with the lockout time of 7 minutes was utilized for postoperative pain relief. For the PCA pump, 60 mg morphine was mixed with 8 mg ondansetron and diluted to 60 ml with normal saline.

Perioperative blood pressure, electrocardiogram, pulse oxygen saturation, end-tidal carbon dioxide concentration, tidal volume, respiratory rate and airway resistance were monitored and symptomatically treated.

### Observation indices

Perioperative indices: Surgical time, incision length, intraoperative blood loss and postoperative hospitalization stay length were observed and recorded.

### Gastrointestinal function indices

The first postoperative anal exhaust time and first defecation time were observed and recorded.

### Pain status

Visual analogue scale was employed for both groups 2 huor, 12 hour and 48 hour after surgery. 0: Pain free; 1-3 points: mild pain; 4-6 points: moderate pain; 7-9 points: severe pain; 10 points: insufferable pain. Meanwhile, the plasma concentrations of lidocaine in the venous blood of the treatment group were measured at the same time points.

### Adverse reactions

Postoperative adverse reactions such as nausea and vomiting, dizziness, headache, subcutaneous emphysema and fat liquefaction of incision were observed and recorded.

### Statistical analysis

All data were analyzed by SPSS 14.0. The categorical data were expressed as mean ± standard deviation and compared by the t test. Inter-group comparisons were performed with analysis of variance. The numerical data were compared by using Chi-square test, and the ordinal data were compared with the Wilcoxon rank-sum test. P<0.05 was considered statistically significant.

## RESULTS

### Perioperative indices

All patients successfully completed the surgery, with similar surgical time, incision length and intraoperative blood loss. However, the postoperative hospitalization stay length of the treatment group was significantly shorter than that of the control group (P<0.05) (Table-II).

**Table-II:** Perioperative indices (x±s).

Group	Case No. (n)	Surgical time (min)	Incision length (cm)	Intraoperative blood loss (ml)	Postoperative hospitalization stay length (d)
Treatment group	39	185.92±33.29	4.39±1.82	67.29±12.76	6.98±2.19
Control group	39	183.55±29.89	4.45±1.72	68.32±11.37	13.76±2.45
t		0.173	0.287	0.429	9.112
P		>0.05	>0.05	>0.05	<0.05

### Indices for gastrointestinal function recovery

The treatment group had significantly shorter first postoperative anal exhaust time and first defecation time than those of the control group (P<0.05) (Table-III).

**Table-III:** Indices for gastrointestinal function recovery (h, x±s).

Group	Case No. (n)	First anal exhaust time	First defecation time
Treatment group	39	65.39±13.29	80.18±16.43
Control group	39	75.20±14.22	91.87±15.00
t		10.983	11.283
P		<0.05	<0.05

### Pain scores

Pain score of the treatment group, which was significantly lower than that of the control group at the postoperative 2 hour (P<0.05), was similar to those of the control group at the postoperative 12 hour and 48 hour. With prolonged time, the score significantly decreased (P<0.05) (Table-IV).

**Table-IV:** Pain scores at different time points (point, x±s).

Group	Case No. (n)	Postoperative 2 h	Postoperative 12 h	Postoperative 48 h
Treatment group	39	2.76±0.56	2.45±0.29	1.71±0.56
Control group	39	3.87±0.67	2.50±0.31	1.81±0.76
t		7.298	0.583	0.981
P		<0.05	>0.05	>0.05

In the meantime, the required lidocaine concentrations of the treatment group were 2.64±1.23 μg/ml, 1.14±0.4 μg/ml and 0.93±0.32 μg/ml respectively 2 hour, 12 hour and 48 hour after surgery.

### Adverse reactions

Postoperative adverse reactions, such as nausea and vomiting, dizziness, headache, subcutaneous emphysema and fat liquefaction of incision, occurred similarly in the two groups, which were alleviated by symptomatic treatment (Table-V).

**Table-V:** Postoperative adverse reactions (n).

Group	Case No. (n)	Nausea and vomiting	Dizziness and headache	Subcutaneous emphysema	Fat liquefaction of incision
Treatment group	39	2	4	2	1
Control group	39	1	3	3	2
Uc		0.193			
P		>0.05			

## DICUSSION

As a common and frequently-occurring disease, cholelithiasis is now being mainly treated by surgeries among which laparoscopic common bile duct exploration has been widely applied. In this study, all patients successfully completed the surgery, and their surgical time, incision length and intraoperative blood loss were insignificantly different.^9^ Although this surgery avoids major traumas and decreases operation steps, patients may suffer from sharp pain because rich nerve endings at the surgical site are stimulated. Meanwhile, they are prone to postoperative adverse reactions in the absence of fulfilling recovery projects, thus posing high requirements for anesthesia and analgesia.^10^ Routine anesthetic agents mainly include long-lasting local anaesthetics, opioid antagonists and non-steroidal anti-inflammatory drugs, of which lidocaine, as an amide local anesthetic, immediately exerts effects after being absorbed in the blood or intravenously administered, with its plasma concentration leveling off after 3-4 hour of continuous intravenous infusion.^11^ Being consistent with the above analysis, the treatment group herein required 2.64±1.23 μg/ml, 1.14±0.4 μg/ml and 0.93±0.32 μg/ml lidocaine respectively 2 hour, 12 h and 48 h after surgery.

Postoperative pain not only torments patients physically and psychologically, but also increases the risk of adverse reactions, while postponing recovery also. Hence, it is of great significance to relieve their pain and emotional stress so as to maintain comfortable recovery, to reduce the incidence rate of perioperative cardiovascular adverse reactions, and to allow early ambulation.^12^ It has previously been reported that perioperative intravenous injection of lidocaine can enhance the postoperative analgesic effect and accelerate early recovery, and that intraoperative continuous infusion can effectively prevent central hyperalgesia through the nociceptive pathway.^13^ In this study, pain score of the treatment group, which was significantly lower than that of the control group at the postoperative 2 hour (P<0.05), was similar to those of the control group at the postoperative 12 hour and 48 hour. With extended time, the pain score significantly decreased (P<0.05). Probably, the pain was milder than that during movement, and normal saline may have local analgesic effect by cooling or diluting intra-articular algogenic substances, leading to similar pain scores of the two groups with elapsed time.^14^ Moreover, by preventing the migration and activation of lymphocytes, lidocaine can mitigate local inflammatory response-induced central hyperalgesia and postoperative pain.^15^

Surgeries are bound to induce traumas and gastrointestinal disorders, but lidocaine is able to reduce the dose of opioids and to directly suppress sympathetic plexuses. In addition, it blocks the sodium channel, thereby inhibiting G protein-coupled receptors, spontaneous pulses of injured dorsal root ganglia and peripheral nerves, as well as pain signal transduction.^16^ In this study, the treatment group had significantly shorter first anal exhaust time and first defecation time than those of the control group (P<0.05), suggesting that lidocaine was beneficial to the recovery of gastrointestinal function.

Notably, long-term use of lidocaine may result in adverse reactions. However, nausea and vomiting, bradycardia and fatigue after intravenous infusion of lidocaine (plasma concentration: 5μg/ml) for five days to treat chronic pain disappear 12 hour after drug discontinuance.^17^,^18^ The two groups herein had similar adverse reactions such as nausea and vomiting, dizziness, headache, subcutaneous emphysema and fat liquefaction of incision, which were alleviated by symptomatic treatment. The outcomes may be attributed to low plasma lidocaine concentration.^19^ Regardless, the plasma concentration, safe dose and administration duration of continuously intravenously infused lidocaine must be further studied.

In summary, laparoscopic common bile duct exploration is a promising minimally invasive surgery for patients with cholelithiasis. Intravenous infusion of lidocaine can rapidly recover the gastrointestinal function and exert short-term analgesic effects, accompanied by mild adverse reactions as well.
